# Supplementary material for: Giant linear strain gradient with extremely low elastic energy in a perovskite nanostructure array
Source: Nat Commun. 2017 Jun 30;8:15994. doi: 10.1038/ncomms15994 (PMC5497064; doi:10.1038/ncomms15994)
Supplement: Supplementary Information [file ncomms15994-s1.pdf]

File name: Supplementary Information
Description: Supplementary Figures, Supplementary Notes and Supplementary References

File name: Peer Review File
Description:

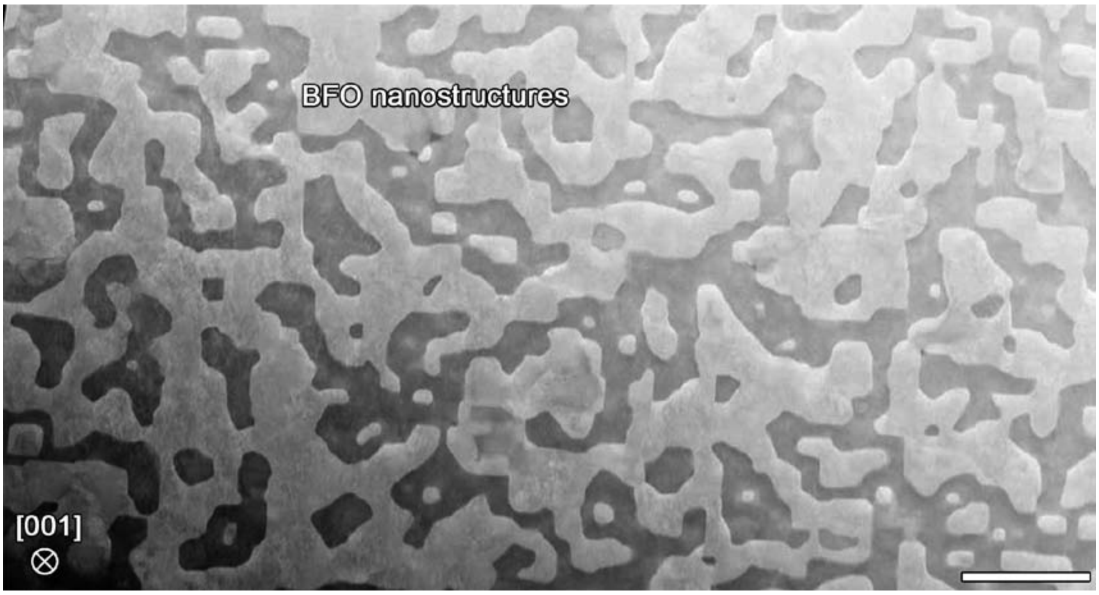

Supplementary Figure 1. Low magnification plane-view HAADF images showing the morphology of the self-assembled $BiFeO_3$ nanostructures. $BiFeO_3$ has larger mean atomic number and shows higher intensity under HAADF imaging mode. Scale bar: 500 nm.

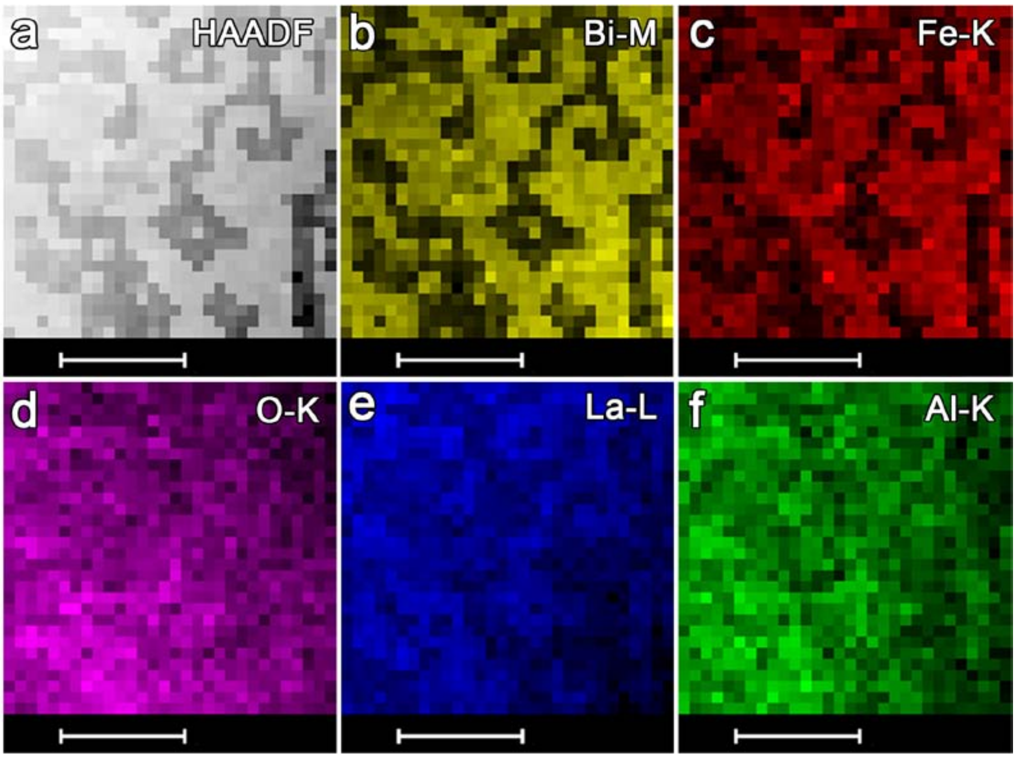

Supplementary Figure 2. Plane-view 2D element mappings of the self-assembled $BiFeO_3$ nanostructures. The $BiFeO_3$ nanostructures can be seen clearly by the EDX (Energy Dispersive X-Ray Spectroscopy) mappings here. Scale bars: 500 nm.

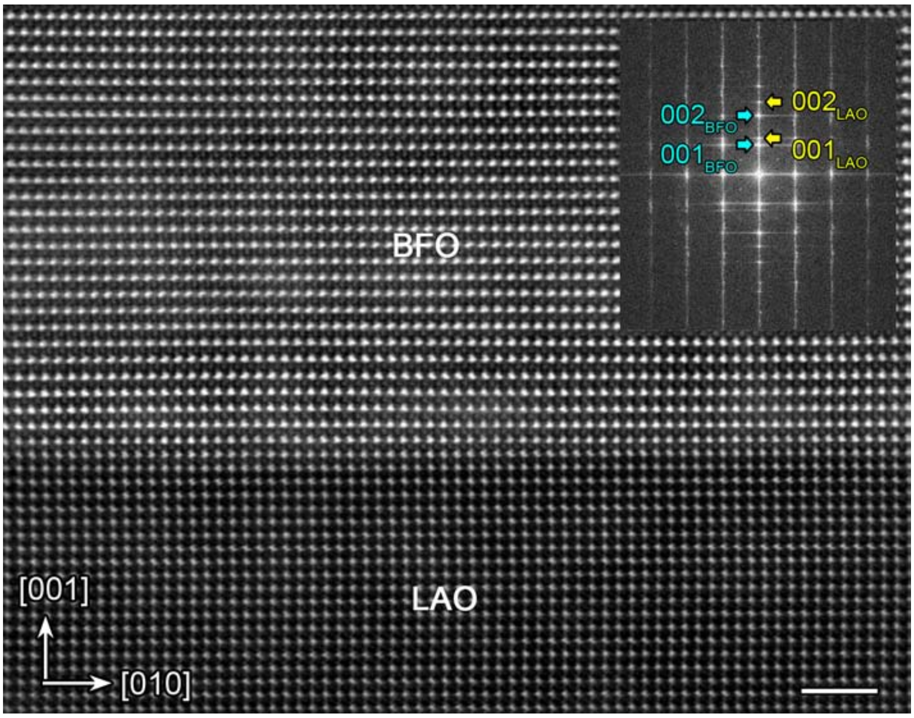

Supplementary Figure 3. A 2D growth of $BiFeO_3$ film on $LaAlO_3$ substrate under a low deposition flux mode. A filtered HAADF-STEM image is shown here. The inset is corresponding Fast Fourier Transform (FFT) pattern of the HAADF-STEM image. Note the famous strain-driven tetragonal BFO phase (T phase) is observed without any interfacial dislocations here. Scale bar: 2nm.

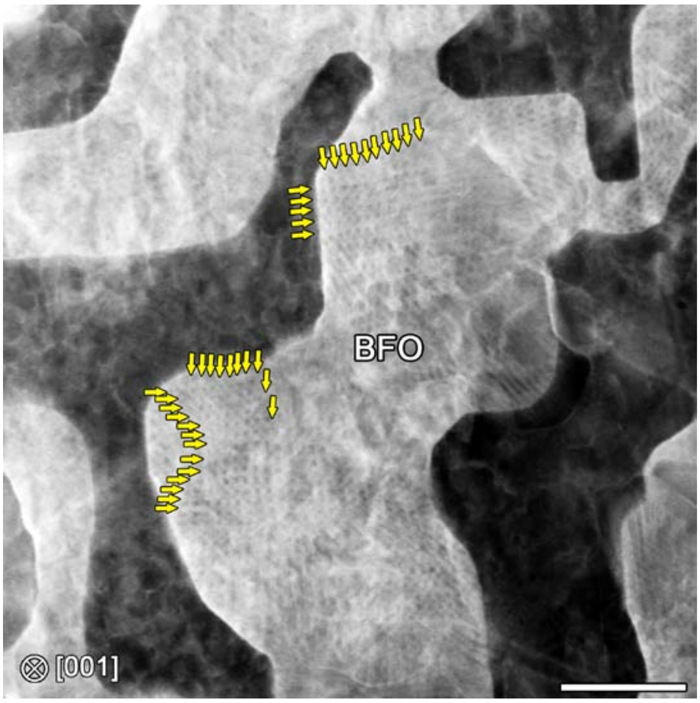

Supplementary Figure 4. Plane-view HAADF-STEM imaging showing the misfit dislocation arrays. Dislocation lines were marked by yellow arrows. Scale bar: 100 nm.

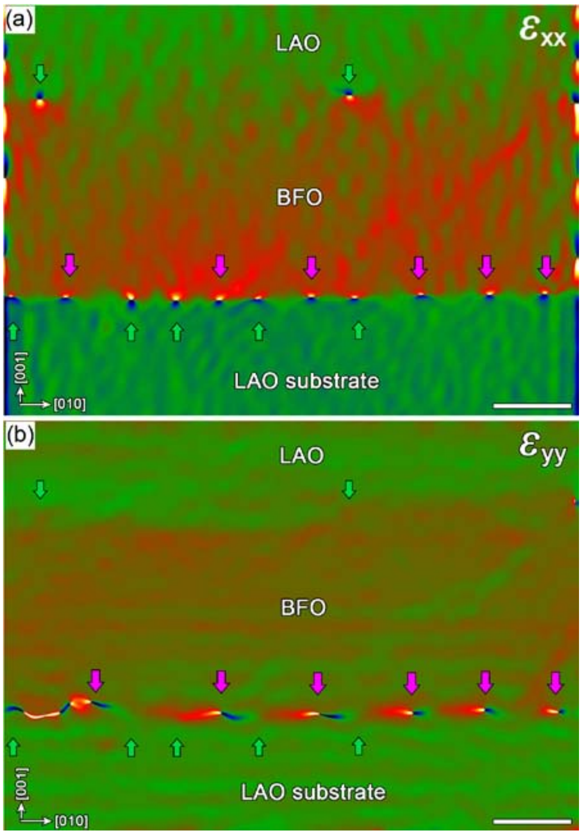

Supplementary Figure 5. Identification of dislocation types via comparisons of in-plane and out-of-plane strain maps. (a), (b), ε_{xx} and ε_{yy} maps via GPA. The Burgers vector of *a*[010] dislocations possesses only in-plane component, while *a*[011] dislocations have both in-plane and out-of-plane components. Thus the dislocation cores which exhibit only out-of-plane strain contrast correspond to *a*[011] dislocations (marked by pink arrows), and the others are *a*[010] dislocations (marked by green arrows). Scale bars: 10 nm.

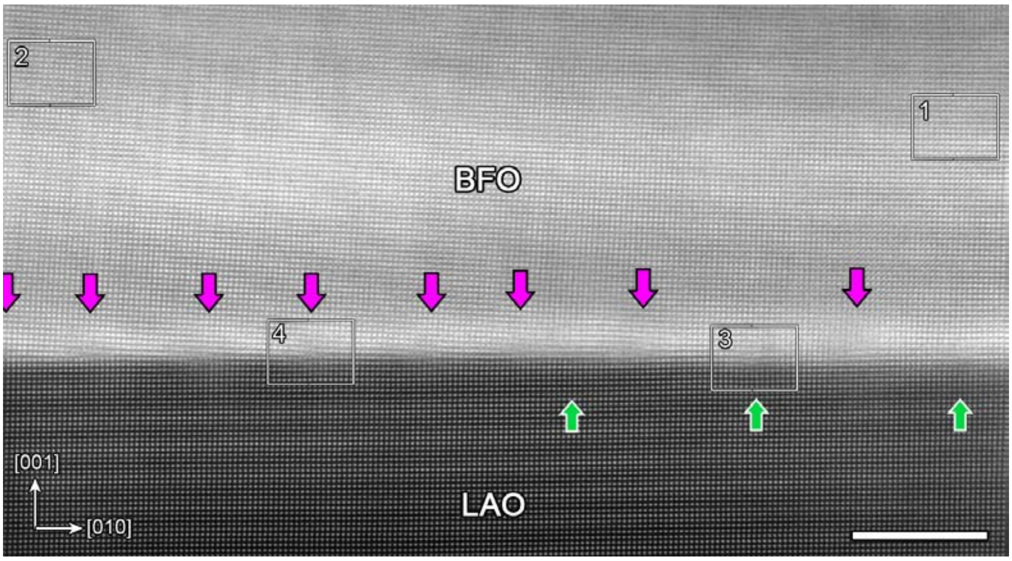

Supplementary Figure 6. A HAADF-STEM image of another $BiFeO_3$ island. Pink and green arrows indicate two different types of interfacial dislocations as that in the main text. Four boxes labeled as 1, 2, 3 and 4 are four typical areas. Noise in the image was reduced by Wiener filter. Scale bar: 10 nm.

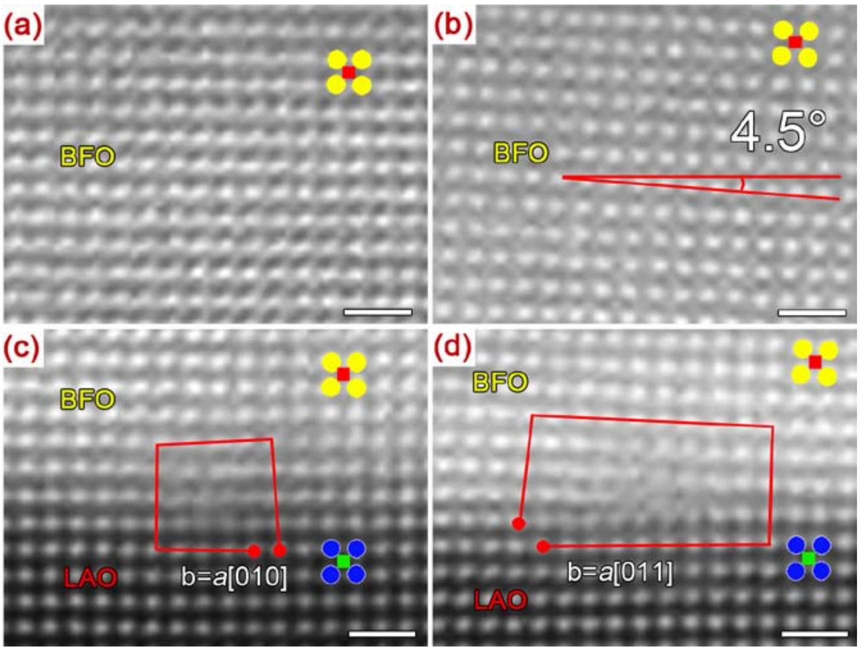

Supplementary Figure 7. Magnified four typical areas in the $BiFeO_3$ island. (a)-(d) correspond to 1, 2, 3 and 4 areas, respectively. Note the relative lattice rotation of (b) compared with (a), which indicates relative lattice rotations of the $BiFeO_3$ island across the in-plane direction. Also note there are two types of interfacial dislocation, as shown in (c) and (d). The insets correspond to the unit-cell schematics of $BiFeO_3$ and $LaAlO_3$ (yellow circles, Bi; red squares, Fe; blue circles, La; green squares, Al). Scale bars: 1 nm.

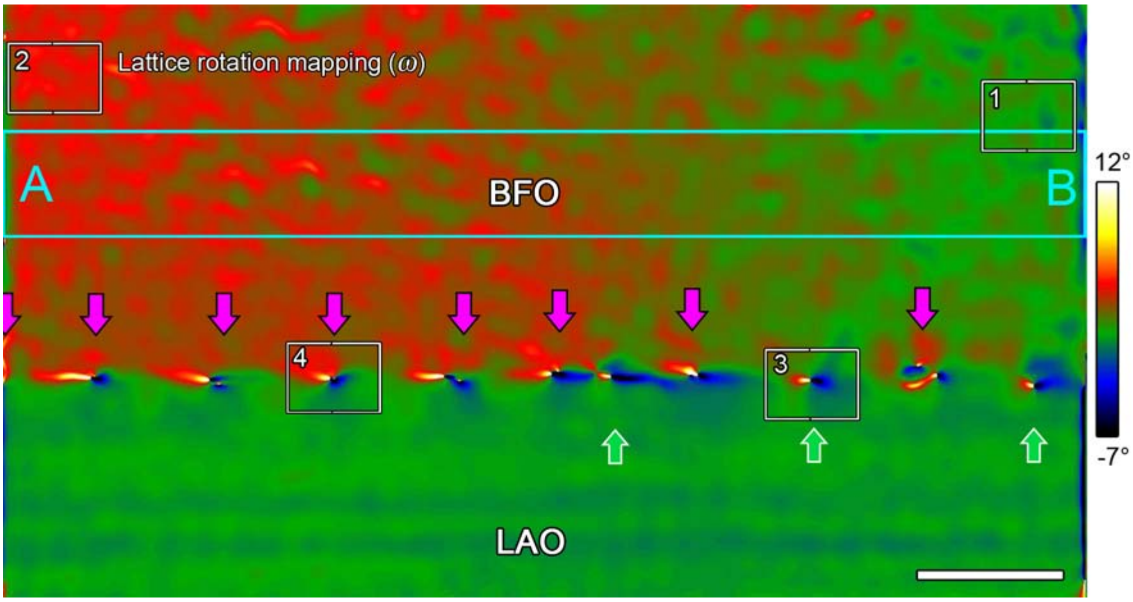

Supplementary Figure 8. Lattice rotation mapping of the $BiFeO_3$ island. Note the continuous change of lattice rotation in $BiFeO_3$ island across the in-plane direction. The blue boxed area labeled as A to B was chosen as a visualization line-profile. Note the obvious contrast at the dislocation cores. Scale bar: 10 nm.

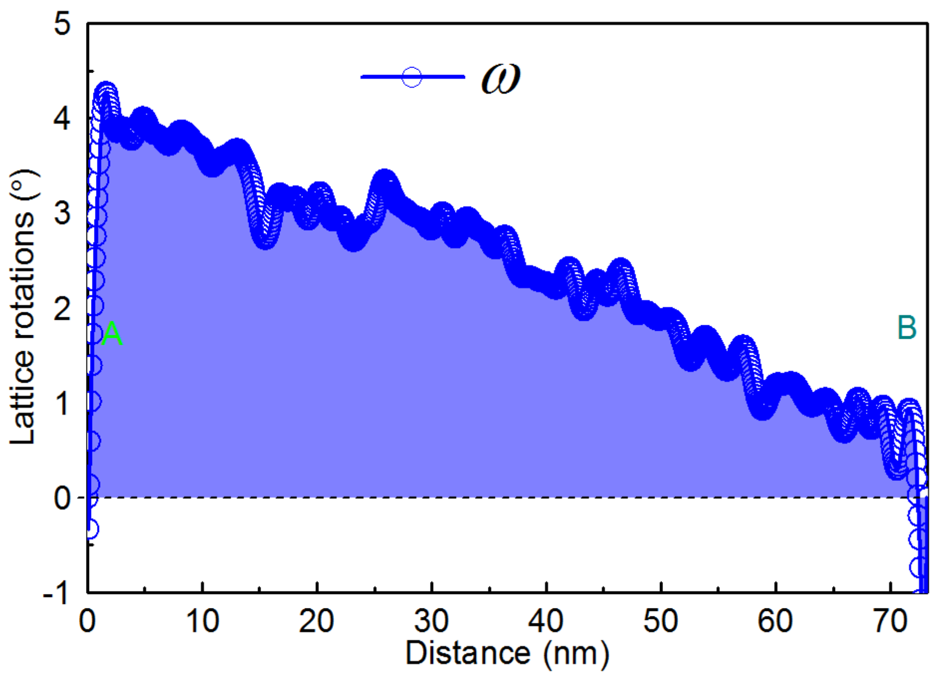

Supplementary Figure 9. Line-profile across A to B in the $BiFeO_3$ island. The continuous decrease of lattice rotation in $BiFeO_3$ island across the in-plane direction can be seen clearly, which changed from about 4.5° at the left to about 0° at the right.

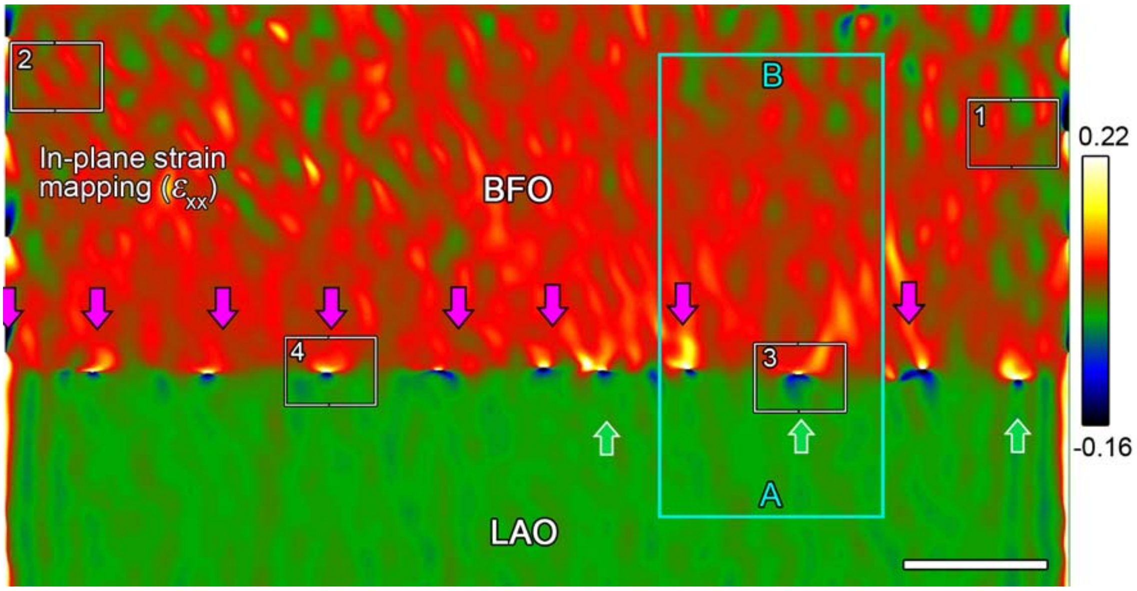

Supplementary Figure 10. In-plane strain mapping (ε_{xx}) of the $BiFeO_3$ island. The blue boxed area labeled as A to B was chosen as a visualization line-profile. The strain effects of different types of dislocations and the corresponding long rang strains are completely the same as shown in the main text. Scale bar: 10 nm.

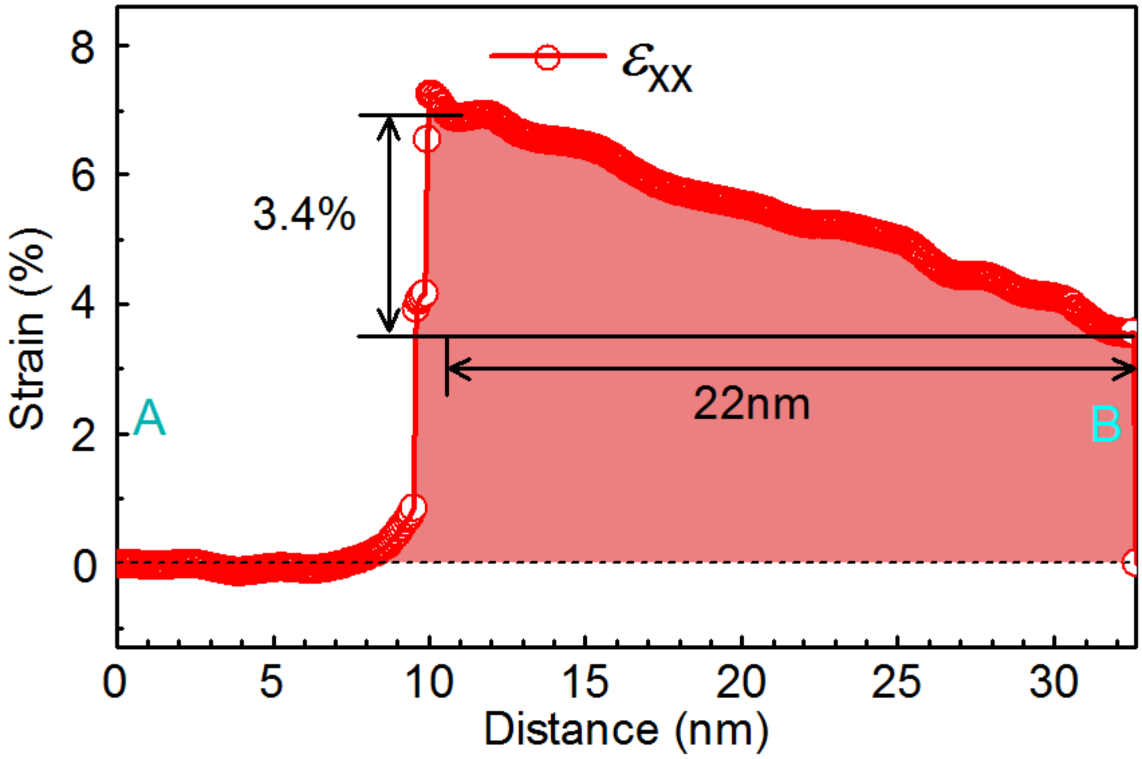

Supplementary Figure 11. Line-profiles of the in-plane strain (ε_{xx}) in the $BiFeO_3$ island. The strain gradient here is also well above $10^6 m^{-1}$ order.

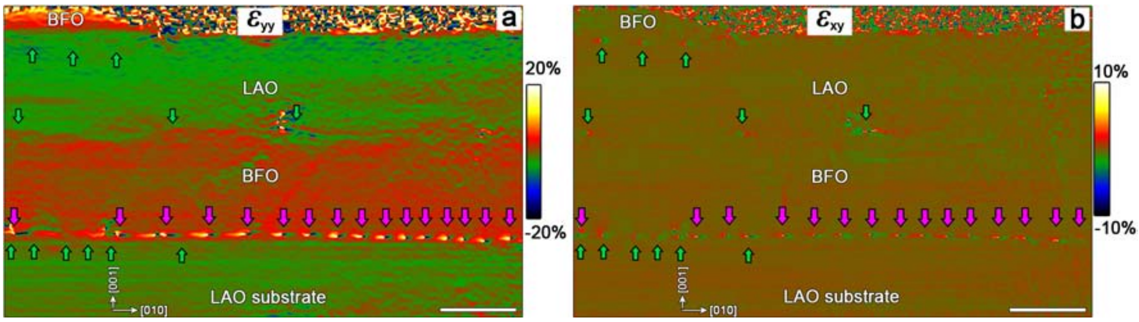

Supplementary Figure 12. Strain mappings of the $BiFeO_3/LaAlO_3/BiFeO_3/LaAlO_3$(001) nanostructure via GPA. (a) Out-of-plane strain mapping (ε_{yy}). (b) Shear strain mapping (ε_{xy}). Note the closely packed *a*[011] dislocations at the right panel of (a), since only the *a*[001] components of *a*[011] dislocations display obvious out-of-plane strain contrast at dislocation cores. Also note the negligible shear strains in the nanostructure in (b), which indicates that the lattice rotation here is rigid-body rotation with little shear strain. Scale bars: 20 nm.

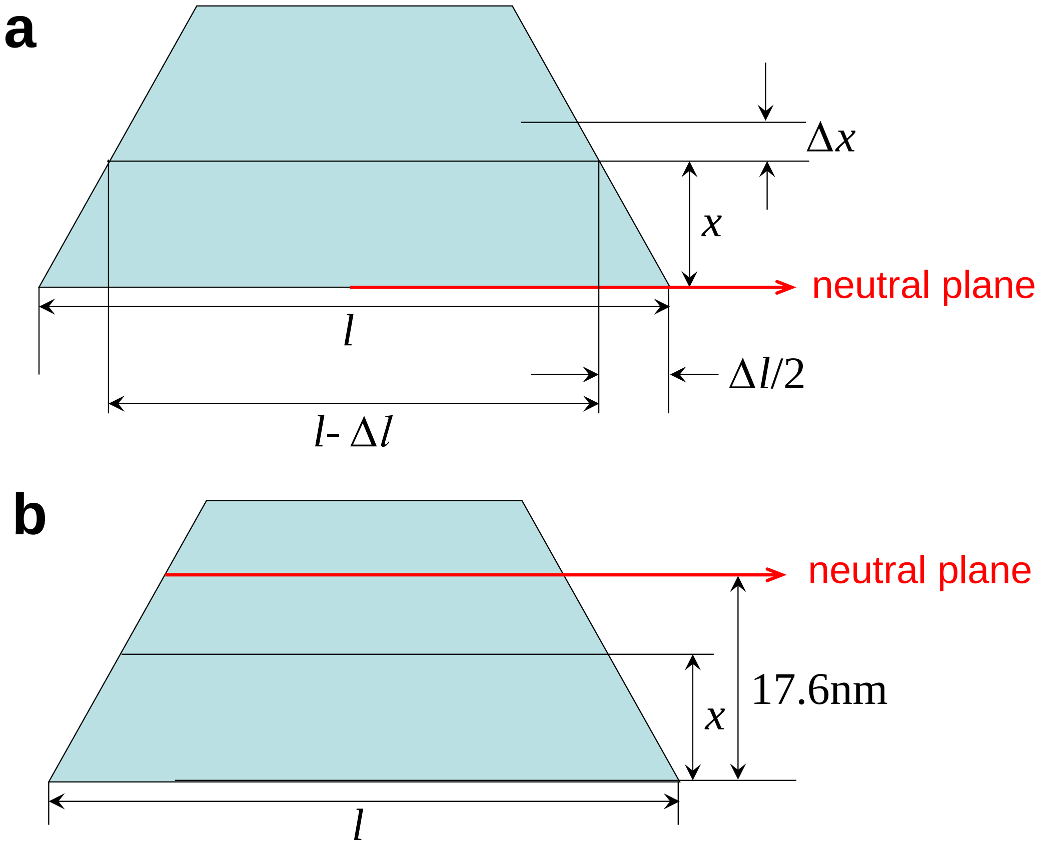

Supplementary Figure 13. Schematics for the calculations of elastic energy of a $BiFeO_3$ nanostructure under linear strain gradient.

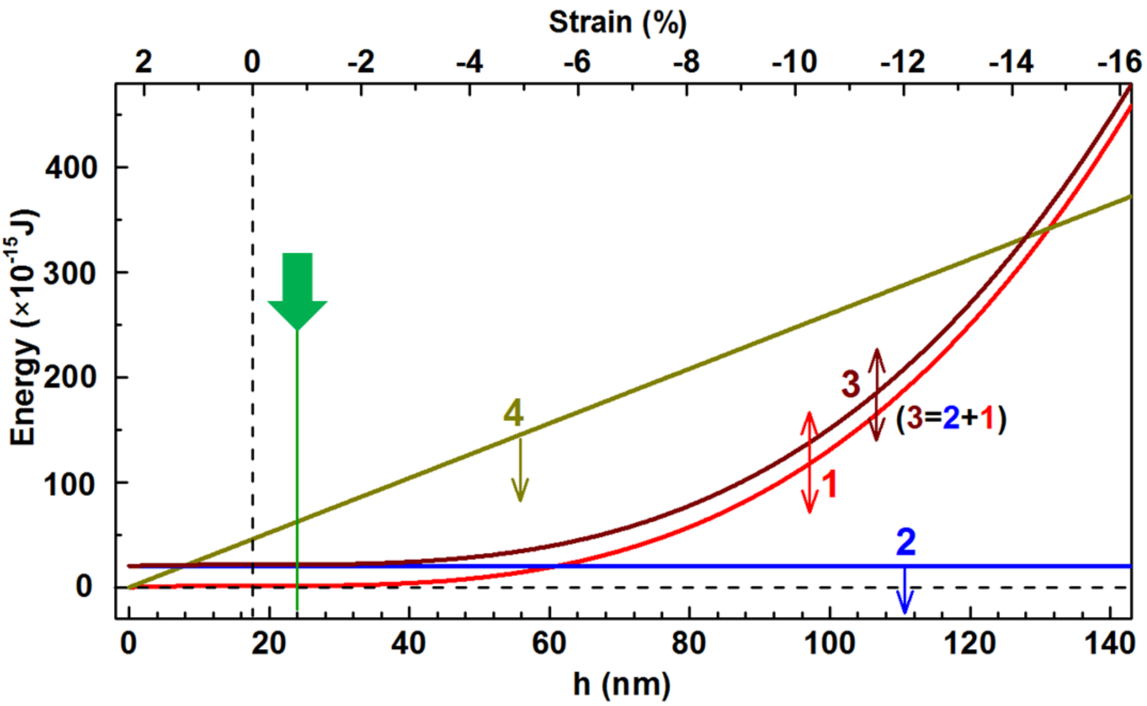

Supplementary Figure 14. Comparisons between elastic energy evolutions for $BiFeO_3$ nanostructures. Linear strain gradient and fully 2D strained state were compared here. Curve 1 is the elastic energy distribution of a $BiFeO_3$ nanostructure under linear strain gradient. Curve 2 is the interfacial dislocation energy of the $BiFeO_3$ nanostructure. Curve 3 is the whole energy of the $BiFeO_3$ nanostructure calculated through 1+2, which represents the energy consumption for producing the experimentally observed disclinations in the $BiFeO_3$ nanostructures. Curve 4 is the elastic energy of a same size $BiFeO_3$ nanostructure under fully 2D strains on the assumption that the $BiFeO_3$ nanostructure is fully strained by the mismatch between $BiFeO_3$ and $LaAlO_3$ (4.5%). The black dotted line indicates the neutral plane where the $BiFeO_3$ lattices are under free strain. The green arrow indicates the thickness termination of the present $BiFeO_3$ nanostructure. It can be seen that, actually, the elastic energy of a hypothetical $BiFeO_3$ nanostructure with thickness smaller than ~60nm is extremely low and can be completely neglected compared with the fully strained one; after ~60nm thickness, the elastic energy increases rapidly. Also note that, if the strain gradient and neutral plane is fixed, then a hypothetical $BiFeO_3$ nanostructure exceeding ~130nm thickness will accumulate elastic energy exceeding the fully coherent one. Thus the ~24nm in present $BiFeO_3$ nanostructure reported here do involve negligible elastic energy.

Supplementary Note 1:

PLD deposition details. $BiFeO_3$ nanostructures were deposited by pulsed laser deposition (PLD), using a Lambda Physik LPX 305i KrF (λ=248 nm) excimer laser. A sintered $BiFeO_3$ ceramic target (3mol% Bi-enriched) and a stoichiometric $LaAlO_3$ ceramic target were used. The target-substrate distance was 40 mm. The background pressure was 10^{-5} Pa. During the growth of the first layer $BiFeO_3$ nanostructures (Supplementary Figure 1 and 2), the substrate temperature was kept at 680℃, with a laser energy density of ~2.0 Jcm^{-2}, a laser repetition rate of 5 Hz and under an oxygen pressure of 11 Pa. The substrate was mechanically polished to expose a fresh substrate surface thus insure a high deposition flux.

During the growth of $LaAlO_3$ layer, the substrate temperature was kept at 680℃, with a laser energy density of ~1.7 Jcm^{-2}, a laser repetition rate of 2 Hz and under an oxygen pressure of 20 Pa. The laser was focused on the ceramic target for 30 min pre-sputtering to stable the $LaAlO_3$ target surface.

During the growth of $BiFeO_3$ layer on top of the $LaAlO_3/BiFeO_3/LaAlO_3$(001) nanostructures, the substrate temperature was kept at 680℃, with a laser energy density of ~1.7 Jcm^{-2}, a laser repetition rate of 2 Hz and under an oxygen pressure of 11 Pa. The laser was focused on the ceramic target for 30 min pre-sputtering to stable the $BiFeO_3$ target surface. The stabilized target allows a lower deposition flux.

Before deposition, all substrates were pre-heated at 750℃ for 5 min to clean the substrate surface and then cooled down to the growth temperature (10℃min^{-1}). After deposition, the samples were annealed at their growth temperature in an oxygen pressure of 5×10^4 Pa for 10 min, and then cooled down to room temperature at a cooling rate of about 5℃min^{-1}. The morphologies of the as grown $BiFeO_3$ nanostructures are shown in Supplementary Figure 1 and 2. The 2D growth of BFO film on LAO substrate under low deposition flux is displayed in Supplementary Figure 3.

Supplementary Note 2.

HAADF-STEM experiment and strain analysis. The samples for the STEM experiments were prepared by slicing, gluing, grinding, dimpling, and finally ion milling. A Gatan PIPS was used for the final ion milling. Before ion milling, the samples were dimpled down to less than 20 μm. The final ion milling voltage was less than 1kV to reduce ion beam damage. HAADF-STEM images were recorded using aberration-corrected scanning transmission electron microscopes (Titan Cubed 60-300kV microscope (FEI) fitted with a high-brightness field-emission gun (X-FEG) and double Cs corrector from CEOS, and a monochromator operating at 300 kV). The beam convergence angle is 25 mrad, and thus yields a probe size of less than 0.10 nm. The diffraction contrast image was recorded using a conventional TEM (Tecnai G2 F30 (FEI) working at 300 kV).

Large scale strain fields were deduced by using custom plugins of GPA under the framework of Gatan DigitalMicrograph software[1]. The GPA is an effective approach to determine strain variations in an atomic HAADF-STEM image[1,2,3]. The visualization of the strains and lattice rotations was carried out using Gatan DigitalMicrograph software.

Supplementary Note 3

Interfacial dislocation arrays as a routine to introduce lattice rotation. The configuration of the interfacial dislocation arrays at the $BiFeO_3/LaAlO_3(001)$ interface can be glanced in Supplementary Figure 4 and 5. Another $BiFeO_3$ nanostructure showing the same strain behaviors as in the main text (Fig. 1-2) was given here, as shown in Supplementary Figure 6-11. The above observed linear strain gradient persevered in lead free perovskite is common phenomenon in the present fast grown $BiFeO_3$ nanostructures on LAO(001) substrate.

The out-of-plane strain (ε_{yy}) and shear strain (ε_{xy}) for the $BiFeO_3/LaAlO_3/BiFeO_3/LaAlO_3(001)$ nanostructure (Fig. 3 in the main text) were shown in Supplementary Figure 12. Note the closely packed *a*[011] dislocations at the right panel of Supplementary Figure 12(a), since only the *a*[001] components of *a*[011] dislocations display obvious out-of-plane strain contrast at dislocation cores.

Supplementary Note 4

Elastic energy consideration of perovskite under linear strain gradient. The elastic energy consumption for producing present observed strain gradients is unexpectedly low (Fig. 5 in the main text). Two types of energies are involved here: 1) the elastic energy consumption and 2) energies of the interfacial dislocation arrays. We consider a $BiFeO_3$ nanoisland with dimension as $l \times t \times h$, where l and t are the in-plane dimensions vertical to and along the imaging direction, respectively (Fig. 1(A) in the main text), h is the out-of-plane thickness. The elastic energy density of such linear strained $BiFeO_3$ island are not affected by the in-plane dimensions, thus we arbitrarily set $l = t = 100$nm. Since the bending deformation of the $BiFeO_3$ island only occurs along a single in-plane [100] direction (l direction, Fig. 1(A) in the main text), thus we infer that along [010] direction (t direction, perpendicular to the observing direction (t) of Fig. 1(A) in the main text), there is no *a*[101] type dislocation and the mismatch along [100] was relaxed by only *a*[100] dislocations. Thus the elastic energy of the bending $BiFeO_3$ island can be calculated as follow, based on an integration for the infinitesimal elastic energy expression ΔUu:

First we consider a $BiFeO_3$ island with dimension as $l \times t \times x$, where x is a variable corresponding to out-of-plane direction (dimension h) here. The elastic energy of a $BiFeO_3$ island under uniaxial deformation Δl along l can be written as[4]:

$$U = \frac{\mathrm{E}tx}{2l}(\Delta l)^2 \tag{1}$$

where E is the elastic modulus. A linear strain gradient deformation is schematically shown in Supplementary Figure 13(a). The linear strain gradient is defined as Δ*S*, thus for an infinitesimal Δ*x* $BiFeO_3$ at *x* (from the neutral plane), the relationship between Δ*S* and corresponding deformation Δ*l* is:

$$\Delta S = \frac{\Delta l / l}{x} \tag{2}$$

thus the elastic energy of this infinitesimal Δ*x* $BiFeO_3$ is Δ*Uu*:

$$\Delta Uu = \frac{\mathrm{E}t\Delta x}{2l}(\Delta Slx)^2 = \frac{\mathrm{E}lt(\Delta S)^2}{2}x^2\Delta x \tag{3}$$

thus the elastic energy of the whole linear strained $BiFeO_3$ is the integration of Δ*Uu* along the *x* direction:

$$Uu = \frac{\mathrm{E}lt(\Delta S)^2}{2}\int_0^x x^2\mathrm{d}x = \frac{\mathrm{E}lt(\Delta S)^2}{2}\frac{x^3}{3} = \frac{\mathrm{E}lt(\Delta S)^2}{6}x^3 \tag{4}$$

note here the elastic energy are calculated from the neutral plane, where the thickness *x* equals 0 (Supplementary Figure 13(a)). Since the neutral plane lies in the (neutral plane at ~17.6nm) $BiFeO_3$ island (Fig. 2(D) in the main text), the function of the elastic energy must be write separately as a piecewise function (Supplementary Figure 13(b)):

$$Uu = \begin{cases} \frac{\mathrm{E}lt(\Delta S)^2}{6}17.6^3 - \frac{\mathrm{E}lt(\Delta S)^2}{6}(17.6 - x)^3, & x \le 17.6 \\ \frac{\mathrm{E}lt(\Delta S)^2}{6}17.6^3 + \frac{\mathrm{E}lt(\Delta S)^2}{6}(x - 17.6)^3, & x > 17.6 \end{cases} \tag{5}$$

using the experimentally observed strain gradient $\Delta S \approx 1.24\times10^6\mathrm{m}^{-1}$, an averaged E ≈ 90 GPa and Poisson ratio ν ≈ 0.3 (refs. 5-7), all the units being normalized to corresponding international unit, the plot of the linear strain energy versus thickness *h* can be drawn as curve 1 in Fig. 5(A) in the main text, or curve 1 in Supplementary Figure 14.

According to Supplementary Equation (5), if the location of neutral plane is a variable (*y*), then the elastic energy consumption distribution versus thickness (*h*) and the location of neutral plane (*y*) of the $BiFeO_3$ nanostructure under linear strain gradient can be write as:

$$Uu = \frac{\mathrm{E}lt(\Delta S)^2}{6}y^3 + \frac{\mathrm{E}lt(\Delta S)^2}{6}(h - y)^3 \tag{6}$$

here *y* < *h*. For a given *h*, the partial derivative of function *Uu* for *y* is:

$$\partial Uu / \partial y = \frac{\mathrm{E}lt(\Delta S)^2}{2}y^2 - \frac{\mathrm{E}lt(\Delta S)^2}{2}(h - y)^2 = \frac{\mathrm{E}lth(\Delta S)^2}{2}(2y - h) \tag{7}$$

thus for a given h, the minimum Uu occurs when $y = h/2$:

$$\partial Uu/\partial y = \frac{\mathrm{E}lth(\Delta S)^2}{2}(2y - h) = 0 \tag{8}$$

According to Supplementary Equation (6), the elastic energy consumption distribution versus thickness (h) and the location of neutral plane (y) of the $BiFeO_3$ nanostructure under linear strain gradient is shown in Fig. 5(c) in the main text. By considering the maximum elastic limit of nanomaterials (~10%, ref. 8), the possible maximum h_m is about 160 nm ($h_m = 2\times10\% / (1.2 \times 10^6 \mathrm{m}^{-1}) \approx 160$ nm, when the neutral plane y is in the middle of h_m). Note that the minimum elastic energy consumption occurs when $y = h/2$, i.e., pure bending.

The interfacial dislocation energy of the dislocation arrays at the $BiFeO_3/LaAlO_3$(001) interface can be estimated by the energy density (energy per unit length of dislocations) of edge dislocation[9]:

$$\Delta U_d \approx \mathrm{G}b^2 \tag{9}$$

it is known[4]

$$\mathrm{G} = \frac{\mathrm{E}}{2(1+\nu)} \tag{10}$$

where G is shear modulus, b is the magnitude of a Burgers vector. For determining the Burgers vectors here, an averaged lattice parameter is needed, since the lattice parameters above the misfit dislocation arrays ($BiFeO_3$ lattice) are different from the lattice below ($LaAlO_3$ lattice). Thus the averaged lattice parameter a_a = (0.3789nm + 0.396nm)/2 = 0.38745nm was used. Thus the Burgers vector for a<100> type dislocations is:

$b_1 = a_a$= (0.3789nm + 0.396nm)/2 = 0.38745nm, and the Burgers vector for a<110> type dislocations is $b_2 = \sqrt{2}\, a_a$ = 0.5479375nm.

As above mentioned, along [010] line direction, there is no a[101] type dislocation and the mismatch along [100] was relaxed by only a[100] dislocations. We consider the mismatch along [100] being fully relaxed by a[100] dislocations, thus the a[100] dislocation spacing can be calculated as S1= $b_1/f \approx$ 8.6nm, where f is the mismatch between $BiFeO_3$ and $LaAlO_3$(4.5%). Along [100] line direction, the numbers of a[011] type dislocations are about twice as the a[010] ones (Supplementary Figure 12). The dislocation spacing for these a[011] or a[010] dislocations are measured as S2 ≈ 6.1nm (Supplementary Figure 12). Thus the dislocation energy of the 2D dislocation arrays is:

$$U_d \approx (t/\mathrm{S1})l\mathrm{G}b_1^{\,2} + \frac{1}{3}(l/\mathrm{S2})t\mathrm{G}b_1^{\,2} + \frac{2}{3}(l/\mathrm{S2})t\mathrm{G}b_2^{\,2} \tag{11}$$

note the dislocation energy is a constant here since the misfit dislocation arrays only exist at the interface. Using Supplementary Equation (11), the plot of the dislocation energy

can be drawn as curve 2 in Fig. 5(A) in the main text, or curve 2 in Supplementary Figure 14. The whole energy consumption for producing present observed strain gradients can thus be determined as 3=1+2 , which was drawn as curve 3 in Fig. 5(A) in the main text, or curve 3 in Supplementary Figure 14.

The elastic energy of a fully 2D strain $BiFeO_3$ island with the same in-plane size ($l \times t \times x$, 100nm×100nm × x) and can be calculated as[10]:

$$U_f = 2lt\mathrm{G}f^2\left(\frac{1+\nu}{1-\nu}\right)x \qquad (12)$$

the elastic energy of a fully strained $BiFeO_3$ nanoisland is straightforward, which increases linearly from zero with thickness h. Using Supplementary Equation (12), the plot of the energy of the fully strain $BiFeO_3$ island versus thickness h can be drawn as curve 4 in Fig. 5(A) in the main text, or curve 4 in Supplementary Figure 14.

Under a mismatch of f = 4.5%, the energy consumption for a fully 2D strained $BiFeO_3$ nanoisland increases extremely rapidly. It is thus obvious that the elastic energy cost for the linear strain is extremely low, especially when the thickness of the $BiFeO_3$ island is small. Thus the observed giant strain gradients here are elastically feasible.

The situation for other perovskite oxides, even other types of functional oxides under such linear strain gradient should exhibit the same negligible elastic energy when compared with a fully 2D strain state with big mismatches, since the elastic constants (elastic modulus E, shear modulus G and Poisson ratio ν) for a specific material do not affect the results obtained in the above formulations.

Supplementary References

1. Hÿtch, M. J., Snoeck, E. & Kilaas, R. Quantitative measurement of displacement and strain fields from HREM micrographs. *Ultramicroscopy* **74**,131–146 (1998).
2. Tang, Y. L. *et al.* Observation of a periodic array of flux-closure quadrants in strained ferroelectric $PbTiO_3$ films. *Science* **348**, 547–551 (2015).
3. Tang, Y. L., Zhu, Y. L. & Ma, X. L. On the benefit of aberration-corrected HAADF-STEM for strain determination and its application to tailoring ferroelectric domain patterns. *Ultramicroscopy* **160**, 57–63 (2015).
4. Beer, F. P., Johnston, E. R., Dewolf J, T. & Mazurek, D. F. *Mechanics of materials, 6th edition*. The McGraw-Hill Companies, Inc. New York, 2012, 226–231 and 694–699.
5. Huang, C. W. *et al.* Abnormal Poisson's ratio and Linear Compressibility in Perovskite Materials. *Adv. Mater.* **24**, 4170–4174 (2012).
6. Huang, C. & Chen, L. Effects of Interfaces on the Structure and Novel Physical Properties in Epitaxial Multiferroic $BiFeO_3$ Ultrathin Films. *Materials* **7**, 5403–5426 (2014).
7. Shang, S. L., Sheng, G., Wang, Y., Chen, L. Q. & Liu, Z. K. Elastic properties of cubic and rhombohedral $BiFeO_3$ from first-principles calculations. *Phys. Rev. B* **80**, 052102 (2009).
8. Li, J., Shan, Z. & Ma, E. Guest Editors, Elastic strain engineering for unprecedented materials properties. *MRS BULLETIN* **39**, 108–114 (2014).
9. Kosevich, A. M. Crystal dislocation and the theory of elasticity, in *Dislocations in Solids*, Vol. 1, North-Holland Pub. Co New York, 1979, 53–54.
10. People, R. & Bean, J. C. Calculation of critical layer thickness versus lattice mismatch for Ge_xSi_{1-x}/Si strained layer heterostructures. *Appl. Phys. Lett.* **47**, 322–324 (1985).
